# Distinct molecular components for thalamic- and cortical-dependent plasticity in the lateral amygdala

**DOI:** 10.3389/fnmol.2014.00062

**Published:** 2014-07-03

**Authors:** Osvaldo Mirante, Federico Brandalise, Johannes Bohacek, Isabelle M. Mansuy

**Affiliations:** ^1^Brain Research Institute, Medical Faculty, University ZürichZürich, Switzerland; ^2^Department of Health Science and Technology, Swiss Federal Institute of TechnologyZürich, Switzerland

**Keywords:** LTD, NR2B, NR2C/D, amygdala, phosphatase, mice, PP1

## Abstract

*N*-methyl-D-aspartate receptor (NMDAR)-dependent long-term depression (LTD) in the lateral nucleus of the amygdala (LA) is a form of synaptic plasticity thought to be a cellular substrate for the extinction of fear memory. The LA receives converging inputs from the sensory thalamus and neocortex that are weakened following fear extinction. Combining field and patch-clamp electrophysiological recordings in mice, we show that paired-pulse low-frequency stimulation can induce a robust LTD at thalamic and cortical inputs to LA, and we identify different underlying molecular components at these pathways. We show that while LTD depends on NMDARs and activation of the protein phosphatases PP2B and PP1 at both pathways, it requires NR2B-containing NMDARs at the thalamic pathway, but NR2C/D-containing NMDARs at the cortical pathway. LTD appears to be induced post-synaptically at the thalamic input but presynaptically at the cortical input, since post-synaptic calcium chelation and NMDAR blockade prevent thalamic but not cortical LTD. These results highlight distinct molecular features of LTD in LA that may be relevant for traumatic memory and its erasure, and for pathologies such as post-traumatic stress disorder (PTSD).

## INTRODUCTION

Synaptic plasticity, a property of neuronal connections characterized by a change in synaptic strength following neuron activation, is essential for memory formation but also for forgetting. Whether presynaptic stimulation increases or decreases synaptic strength depends on the magnitude of post-synaptic calcium elevation (Citri and Malenka, 2008). Long-term potentiation (LTP), a form of synaptic strengthening, is induced by a high rise in intracellular calcium concentration leading to activation of protein kinases. In contrast, long-term depression (LTD), a form of synaptic weakening, requires a moderate rise of intracellular calcium concentration that activates protein phosphatases including PP2B (calcineurin) and subsequently PP1 (Mulkey et al., 1993, 1994; Jouvenceau et al., 2003, 2006; Pi and Lisman, 2008). Once activated, PP1 dephosphorylates some of its targets in synaptic terminals (Morishita et al., 2001), in particular, post-synaptic NMDAR and AMPAR subunits, leading to NMDAR downregulation and AMPAR endocytosis, ultimately resulting in synaptic depression [for review, see (Mansuy and Shenolikar, 2006)].

In the lateral amygdala (LA), LTP is associated with the formation of fear memory (McKernan and Shinnick-Gallagher, 1997; Rogan et al., 1997; Tsvetkov et al., 2002), while LTD is thought to underlie the extinction of fear memory (Kim et al., 2007; Hong et al., 2009; Park et al., 2012). Molecular manipulations that interfere with fear extinction do indeed impair LTD (Lin et al., 2003a,b; Dalton et al., 2008, 2012; Ryu et al., 2008). The LA is a complex limbic structure that integrates sensory information from cortical and thalamic afferents. These afferents are highly plastic (Pape and Pare, 2010; Johansen et al., 2011) and converge onto single neurons in LA (Humeau et al., 2005). To date, LTD in LA has been mostly studied at the thalamic pathway, essentially because it is easier to induce than at the cortical pathway (Heinbockel and Pape, 2000; Albrecht, 2007; Tchekalarova and Albrecht, 2007). Similar to fear extinction (Falls et al., 1992; Sotres-Bayon et al., 2007; Liu et al., 2009; Dalton et al., 2012), LTP at the thalamic pathway depends on NMDARs and is primarily associated with the NR2B subunit (Wang and Gean, 1999; Sotres-Bayon et al., 2007; Müller et al., 2009; Yu et al., 2010; Dalton et al., 2012). In contrast, the mechanisms of LTD at the cortical pathway remain unknown, but are postulated to be different from those at the thalamic pathway (Doyere et al., 2003; Humeau et al., 2003). We investigated these mechanisms in adult mouse LA and examined whether they involve the phosphatases PP2B and PP1, and which NMDAR subunits they recruit. Here we show that both PP2B and PP1 are involved in LTD in the amygdala, but that distinct NMDAR subunits are implicated at thalamic and cortical pathways. While LTD depends on NR2B-containing NMDARs at the thalamic pathway, it requires NR2C/D-containing NMDARs at the cortical pathway. We also show that LTD is induced post-synaptically at the thalamic pathway, but not at the cortical pathway.

## MATERIAL AND METHODS

### ANIMALS

For all experiments, adult male mice C57Bl/6 (8–12 weeks old) were used. Animals were housed in standard housing conditions in a temperature- and humidity-controlled facility on a 12 h reversed light/dark cycle. Mice had free access to food and water. All procedures were carried out in accordance with the guidelines of the Veterinary Office of the Canton of Zurich, Switzerland, and approved by its Commission for Animal Research (License numbers 150/2006 and 105/2008).

### SLICES PREPARATION

Mice were anesthetized with isoflurane 99.9% (AttaneTM) and rapidly decapitated. Immediately after decapitation, the brain was extracted and sectioned in coronal slices (400 μm thick for extracellular field recordings, 300 μm for whole-cell patch clamp recordings) in ice-cold modified artificial cerebrospinal fluid (aCSF) containing 175 mM sucrose, 20 mM NaCl, 3.5 mM KCl, 1.25 mM NaH2PO4, 26 mM NaHCO3, 1.3 mM MgCl_2_, and 11 mM D-(+)-glucose, and gassed with 95% O_2_/5% CO_2_ using a vibratome (VT 1000S; Leica Microsystems, Bannockburn, IL, USA). Coronal slices were placed in a holding chamber at 34^∘^C and incubated in normal aCSF containing 119 mM NaCl, 2.5 mM KCl, 1.3 mM NaH_2_PO_4_, 26 mM NaHCO_3_, 1.3 mM MgCl_2_, 2.5 mM CaCl_2_, and 11 mM D-(+)-glucose, and continuously bubbled with 95% O_2_/5% CO_2_ at 34^∘^C for at least 2.5 h, prior to recording. For recording, slices were transferred to a superfusion (1.5–2.5 ml/min flow rate) chamber (Warner Instruments) heated at 33.5-34^∘^C and held below a platinum wire.

### ELECTROPHYSIOLOGY

**FIGURE 1:**
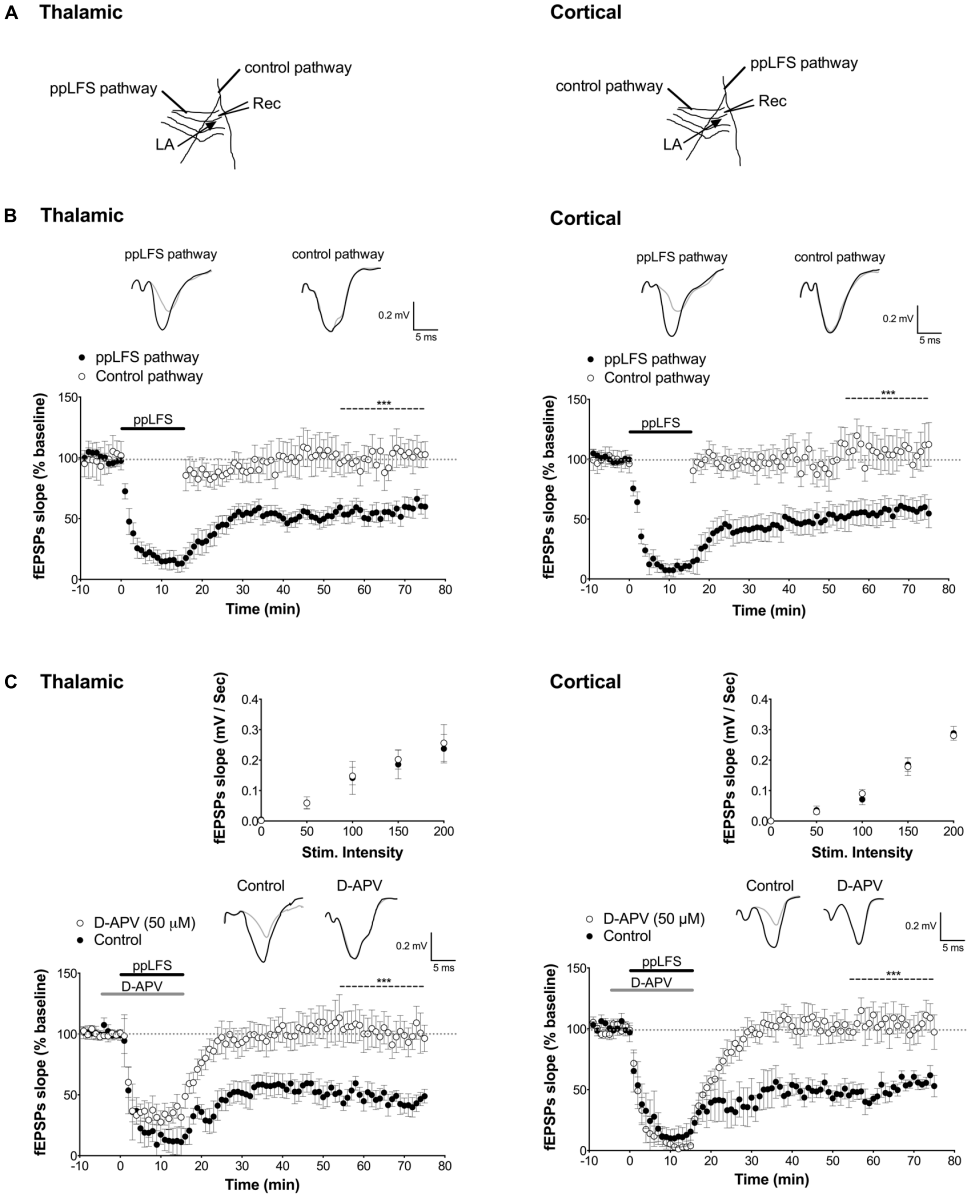
**Input-specific NMDAR-dependent LTD at the thalamic and cortical pathways in the lateral amygdala. (A)** Schematic illustration of electrode placement for ppLFS and control pathway recording of the thalamic pathway (left) and the cortical pathway (right). **(B)** Robust, long-lasting LTD was specifically induced at the pathway receiving ppLFS (thalamic, *n* = 18; cortical, *n* = 18) but not at the control pathway (thalamic: *n* = 11; cortical: *n* = 14). Insets show representative traces of extracellular field potentials averaged across 10 min before ppLFS (black line) and the last 10 min of recording after ppLFS (gray line). **(C)** D-APV (50 μM) prevents LTD at thalamic afferents (control: *n* = 6; D-APV: *n* = 9) and at cortical afferents (control: *n* = 5; D-APV: *n* = 9). Insets show I/O curves on top and below representative traces of extracellular field potentials averaged across 10 min before ppLFS (black line) and the last 10 min of recording after ppLFS (gray line). Data represent mean ± SEM. ***p < 0.001.

The recording electrode was placed in the dorsal part of the LA, and the stimulation electrodes were placed close to the internal capsule and externally to the capsule to stimulate fibers originating from the thalamus or auditory cortex, respectively (see **Figure 1A**). Extracellular field excitatory post-synaptic potentials (fEPSPs) were recorded from the dorsal part of the LA, while basal single-electrical stimuli at 0.05 Hz were applied at both pathways. After 10 min of stable baseline fEPSPs recording, paired pulse low-frequency stimulation [ppLFS, 900 pulses at 1 Hz, interstimuli interval (ITI) of 40 ms] was used to induce LTD (Müller et al., 2009). To test input specificity, ppLFS was induced at only one pathway (ppLFS pathway) whereas the other pathway was used as control and was stimulated with 0.05 Hz baseline stimulation. fEPSPs were recorded using a glass pipette (2–4 MΩ of resistance) filled with normal aCSF. An input/output (I/O) response curve was established by varying the intensity of single-pulse stimulation. The stimulus intensity that evoked a fEPSP equal to 50% of the maximum response was used for all stimulations. fEPSPs were amplified (Multiclamp 700B), filtered (low-pass filter 1 kHz, high-pass filter 1 Hz) and digitized at 10 kHz (Axoclamp 10.2). Whole-cell recordings were performed in a blind approach (Castaneda-Castellanos et al., 2006). The patch pipette (4–8 MΩ resistance) was filled with a solution containing (in mM): potassium gluconate 126, NaCl 4, MgSO4 1, BAPTA-free 0.1, BAPTA-Ca^2+^ 0.05, glucose 15, ATP 3, HEPES 5 (pH was adjusted to 7.2 with KOH) and GTP 0.1. Membrane potential was measured relative to an agar-bridge reference electrode. Reported membrane potential values were adjusted off-line for liquid-junction potentials (usually <5 mV). Voltage-clamp mode was used to record evoked excitatory post-synaptic currents (eEPSCs) from thalamic and cortical pathways. After stable baseline recording for at least 10 min, ppLFS stimulation was delivered in current-clamp configuration. Before and after ppLFS, series resistance was monitored by measuring the passive current transients induced by 10 mV hyperpolarizing voltage steps from a holding potential of -60 mV. Accepted deviations from this parameter in current transients recorded over the time-windows used for statistical analysis were <10% (Brandalise et al., 2012). Data were recorded using an Axopatch 200B amplifier, sampled with a Digidata-1440 interface (sampling time = 250 msec for current-clamp recording, 10 ms for voltage-clamp recordings) and analyzed with P-CLAMP software (Axon Instruments, Foster City, CA, USA) and Origin software (Microcal Software, Northhampton, MA, USA).

### DRUG APPLICATION

All drugs were bath applied at the indicated concentration starting at least 45 min before ppLFS and throughout recording, except D-(-)-2-Amino-5-phosphonopentanoic acid (D-APV, 50 μM, Tocris), which was perfused for 10 min, starting 5 min prior to ppLFS delivery. To block specific NMDAR subunits, the NR2B antagonists ifenprodil hemitartrate (10 μM, Tocris) and Co101244 (1 μM, Tocris) were used, and the NR2C/D-antagonist [±]-*cis*-1-[phenanthren-2yl-carbonyl]piperazine-2,3-dicarboxylic acid (PPDA, Tocris, 0.25 μM to preferentially block NR2C/D-containing receptors and 1 μM to block NR2 subunits nonspecifically). FK-506 (100 μM, Tocris) and tautomycetin (4 nM, Tocris) were used to antagonize PP2B and PP1 activity, respectively (Mitsuhashi et al., 2001; Jouvenceau et al., 2003). The calcium chelator 1,2-bis(*o*-aminophenoxy)ethane-*N,N,N*^′^*,N*^′^-tetraacetic acid (BAPTA, 100 mM, Tocris) and the NMDAR open-channel blocker MK-801 (Dizocilpine, 40 μm, Tocris) were dialysed in individual post-synaptic LA neurons for >10 min through the patch pipette. To specifically and fully block activated NMDARs during MK-801 dialysis, cells were progressively depolarized from the holding potential of -70 mV to +30 mV, while thalamic or cortical pathways were stimulated about 200–300 times to allow irreversible binding of MK-801 to activated post-synaptic NMDARs (Humeau et al., 2003; Yang et al., 2008). Consequently, the post-synaptic NMDAR component of EPSC activity was reduced after MK801 dialysis (charge transfer reduced by 28.6 ± 9.5% *n* = 3 for the thalamic pathway, and 17.2 ± 6.7% *n* = 3 for the cortical pathway). Cells were clamped again at -70 mV for another 10 min showing no significant change in the peak amplitude of AMPAR-mediated responses.

### DATA ANALYSIS

Data analysis was performed using Clampfit software (v10.2, Molecular Devices, Sunnyvale, DA, USA), GraphPad Prism (GraphPad Software Inc., San Diego, CA, USA), and Excel (Microsoft). For all recordings, fEPSP slope, and EPSP and EPSC amplitude were normalized to the average of baseline slope and amplitude, respectively. To improve the signal-to-noise ratio, data were averaged into 1 min bins. For each experiment, two to three slices per animal were recorded, one was always used as control slice and one or two slices received drug-treatment. For statistical analyses, individual animals (not slices) were considered biological replicates. For both extracellular field and whole-cell recordings, data are expressed as mean ± SEM. Statistical comparisons were performed using Student’s unpaired *t*-tests when two groups were compared. One-way ANOVAs were used when more than two groups were compared. If significant, ANOVAs were followed using Duncan’s *post hoc* test. Significance was set to *p* < 0.05.

**FIGURE 2:**
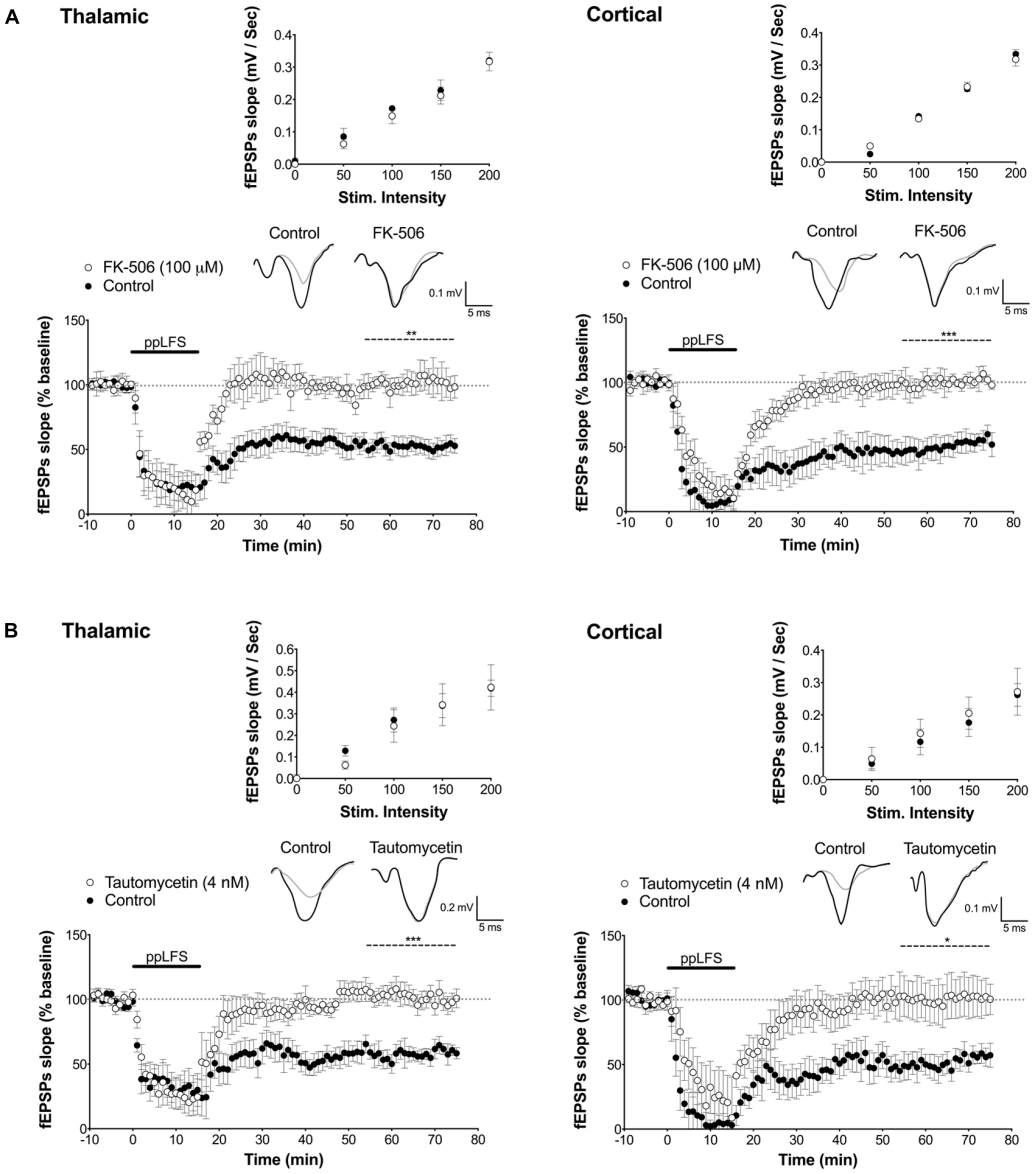
**PP2B and PP1 involvement in LTD at the thalamic and cortical pathways in the lateral amygdala. (A)** The PP2B antagonist FK-506 (100 μM) blocks LTD induced at thalamic afferents (left panel, control: *n* = 5; FK-506: *n* = 5) and at cortical afferents (right panel, control: *n* = 5; FK-506: *n* = 5). **(B)** The PP1 antagonist tautomycetin (4 nM) blocks LTD induced at thalamic afferents (control: *n* = 5; tautomycetin: *n* = 10), and at cortical afferents (control: *n* = 7; tautomycetin: *n* = 8). Insets show I/O curves on top and below representative traces of extracellular field potentials averaged across 10 mins before ppLFS (black line) and the last 10 min of recording after ppLFS (gray line). Data represent mean ± SEM. ***p < 0.001, **p < 0.001, *p < 0.05.

**FIGURE 3:**
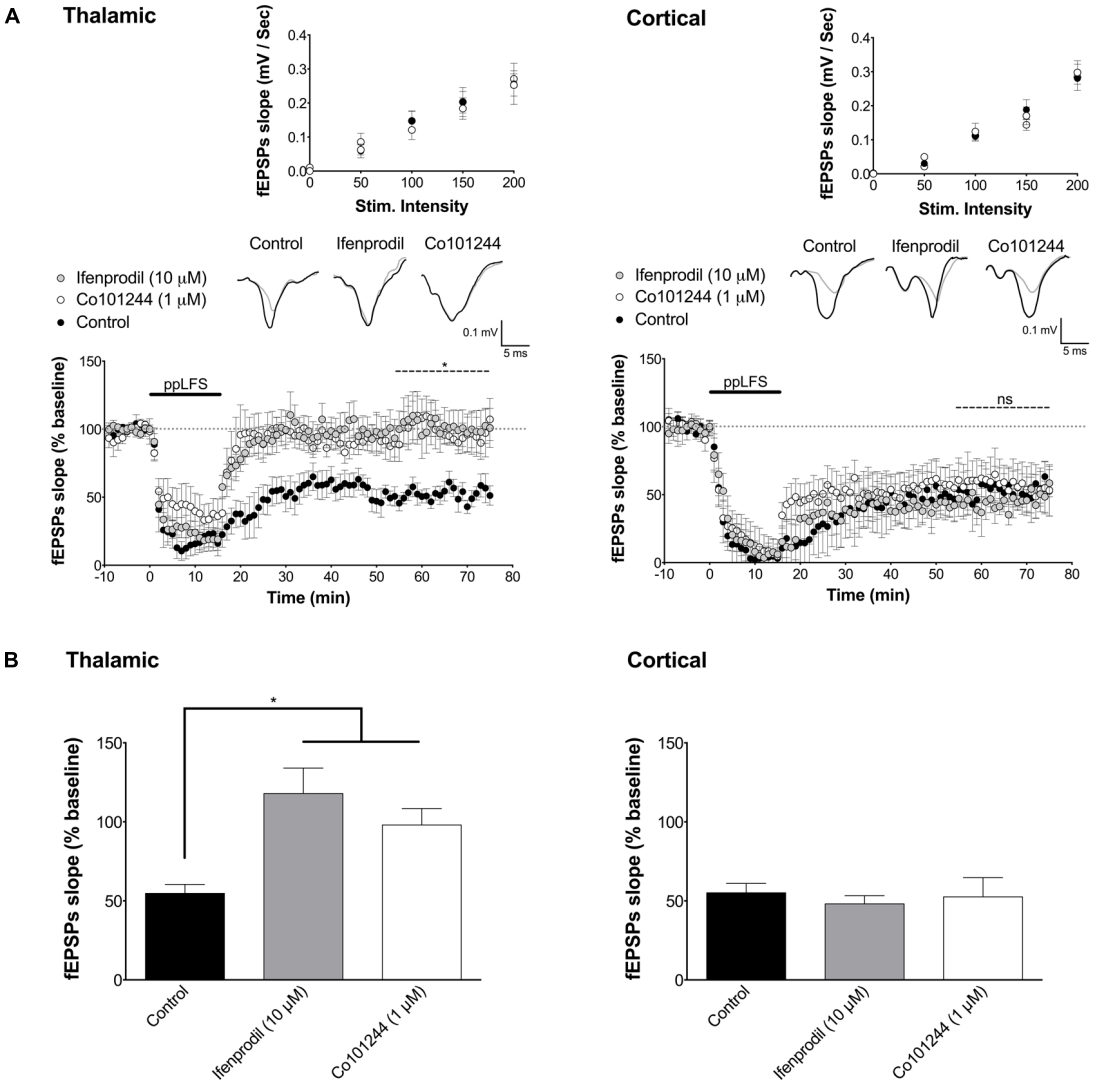
**LA–LTD at thalamic inputs specifically depends on NR2B-containing NMDARs. (A)** Ifenprodil (10 μM) and Co101244 (1 μM) block LTD at thalamic afferents (left panel, control: *n* = 5; ifenprodil: *n* = 8; Co101244: *n* = 5) but not at cortical afferents (right panel, control: *n* = 5; ifenprodil: *n* = 6; Co101244: *n* = 5). **(B)** Summary of the average fEPSP slope over the last 20 min of recording after ppLFS. Insets show I/O curves on top and below representative traces of extracellular field potentials averaged across 10 min before ppLFS (black line) and the last 10 min of recording after ppLFS (gray line). Data represent mean ± SEM. *p < 0.05, ns = non significant.

## RESULTS

### PATHWAY-SPECIFIC LTD IN LA

Using extracellular field recording, we first assessed whether a paired-pulse low frequency stimulation protocol (ppLFS) induces stable and input-specific LTD at thalamic and cortical afferents to the LA in slices from adult mouse (for electrode placement see **Figure 1A**). A robust LTD that lasted over 1hr was specifically induced at the pathway receiving ppLFS but not at a control pathway, both at thalamic (ppLFS pathway: 53.3 ± 4.3%, *n* = 18 vs. control pathway: 106.2 ± 10.9%, *n* = 11, *p* < 0.001, **Figure 1B**) and cortical input (ppLFS pathway: 52.7 ± 3.6%, *n* = 18 vs. control pathway: 108.6 ± 12.2%, *n* = 14, *p* < 0.001, **Figure 1B**). The magnitude of fEPSP suppression was comparable between thalamic and cortical ppLFS (*p* > 0.9). These results indicate that the ppLFS protocol leads to a strong and input-specific induction of LTD (LA–LTD) at both thalamic and cortical pathways to LA.

### NMDAR-DEPENDENT LTD IN LA DEPENDS ON PROTEIN PHOSPHATASES

In the hippocampus, the most common form of LTD requires post-synaptic rise in calcium that depends on NMDARs, and is associated with activation of a PP2B/PP1 signaling cascade (Collingridge et al., 2010). Both PP2B and PP1 are known to be negative regulators of plasticity that further, can act as memory suppressors (Mansuy et al., 1998; Malleret et al., 2001; Genoux et al., 2002). We thus first tested whether LA–LTD is NMDAR-dependent at both pathways using extracellular field recordings. LTD was fully blocked by the NMDAR antagonist D-APV (50 μM) at both, the thalamic (control: 50.6 ± 5.6%, *n* = 6; D-APV: 103.0 ± 10.4%, *n* = 9, *p* < 0.001, **Figure 1C**) and cortical (control: 53.7 ± 2.9%, *n* = 5; D-APV: 114.8 ± 9.9%, *n* = 9, *p* < 0.001, **Figure 1C**) pathway, demonstrating that LA–LTD depends on NMDARs at both pathways. Input/output (I/O) curves were not affected by D-APV, suggesting that basal synaptic transmission was not altered (**Figure 1C**, insets). Next, we examined whether PP2B and PP1 are involved in LA–LTD. Perfusion of the selective PP2B inhibitor FK-506 (100 μM) abolished LTD at both, thalamic (control: 54.9 ± 1.9%, *n* = 5; FK-506: 102.4 ± 10.9%, *n* = 5, *p* < 0.01, **Figure 2A**) and cortical (control: 48.3 ± 2.5%, *n* = 5; FK-506: 96.0 ± 5.0%, *n* = 5, *p* < 0.001, **Figure 2A**) pathways. Similarly, bath application of the specific PP1 inhibitor tautomycetin (4 nM) abolished LA–LTD at both pathways (Thalamic, control: 57.5 ± 5.2%, *n* = 5; tautomycetin: 101.4 ± 5.9%, *n* = 10, *p* < 0.001. Cortical, control: 52.4 ± 6.7%, *n* = 7; tautomycetin: 118.2 ± 20.8%, *n* = 8, *p* < 0.05, **Figure 2B**). I/O curves were not affected by FK-506 (**Figure 2A**, insets) or tautomycetin (**Figure 2B**, insets), suggesting that basal synaptic transmission was not altered. These results show that LA–LTD requires PP2B and PP1 at both thalamic and cortical pathways.

### LA–LTD DEPENDS ON ACTIVATION OF DIFFERENT NR2 SUBUNITS AT THALAMIC AND CORTICAL AFFERENTS

We next investigated the NMDAR subunit composition implicated in LA–LTD at both inputs. While NR2A-containing receptors have previously been suggested to be involved in LTP in different brain structures, NR2B-containing receptors are thought to be involved in LTD (Liu et al., 2004; Massey et al., 2004; Bartlett et al., 2007; Banerjee et al., 2009; Dalton et al., 2012), particularly in LA–LTD at the thalamic input (Yu et al., 2010; Dalton et al., 2012). At the cortical pathway, however, the NMDAR subunit composition is still unclear (Weisskopf and LeDoux, 1999; Müller et al., 2009). To test whether NR2B is required for LTD at both pathways, we used the selective NR2B antagonists ifenprodil (10 μM) and Co101244 (1 μM). While both antagonists fully blocked LTD at the thalamic pathway (control: 54.7 ± 5.7%, *n* = 5; ifenprodil: 118.0 ± 16.0%, *n* = 8; Co101244: 98.0 ± 10.5%, *n* = 5, *p* < 0.05 in both cases, **Figure 3**), they had no effect on LTD at the cortical pathway (control: 55.2 ± 5.9%, *n* = 5; ifenprodil: 48.2 ± 5.2%, *n* = 6; Co101244: 52.5 ± 12.2%, *n* = 5, *p* > 0.8, **Figure 3**). Ifenprodil and Co101244 did not affect I/O curves, suggesting no effect on basal synaptic transmission (**Figure 3A**, insets). These results demonstrate that LTD at the thalamic pathway is NR2B-dependent, while LTD at the cortical pathway is not.

**FIGURE 4:**
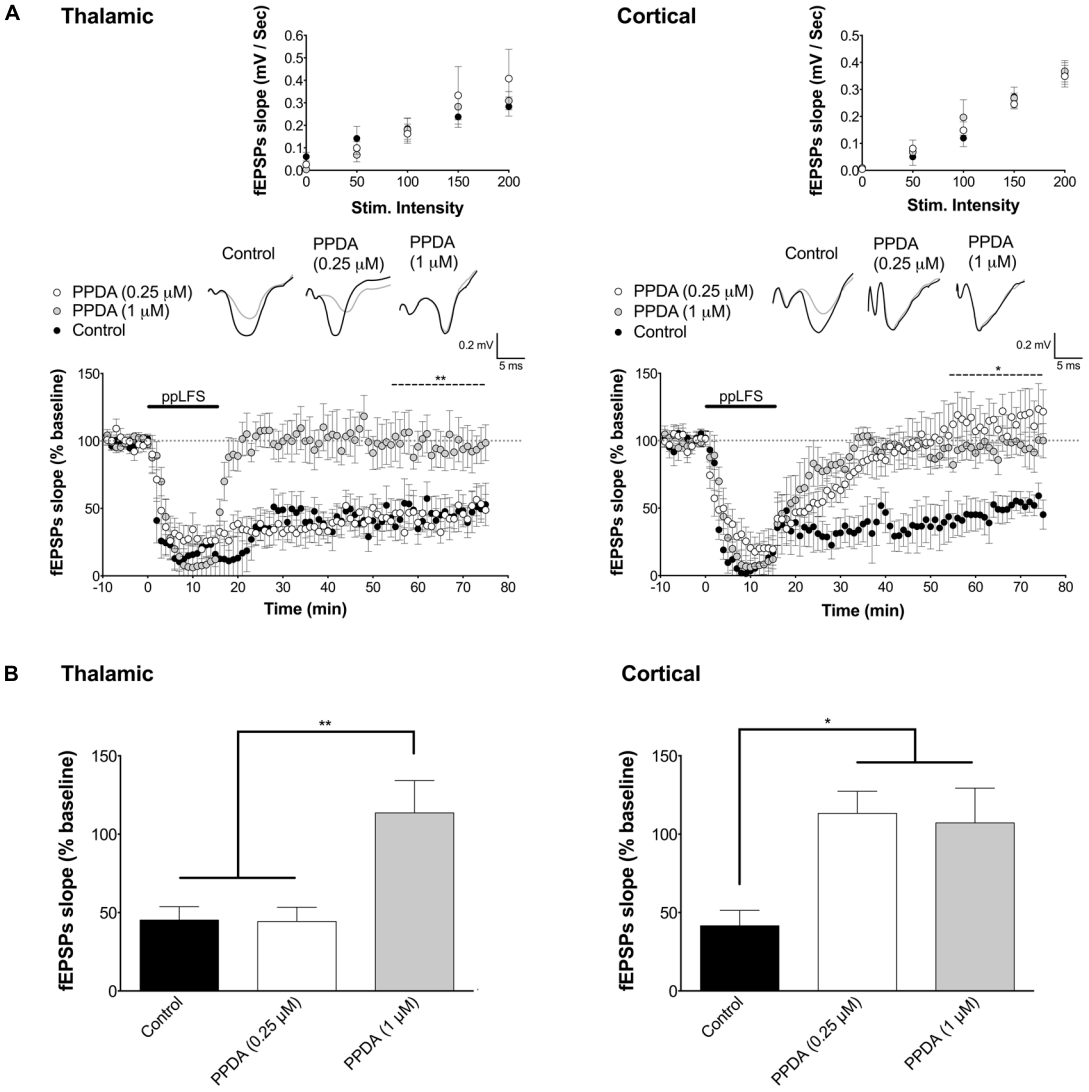
**LA–LTD at thalamic inputs specifically depends on NR2C/D-containing NMDARs. (A)** PPDA does not affect LTD at the thalamic pathway at the NR2C/D-selective low dose of 0.25 μM, but blocks LTD at the non-selective high dose of 1 μM (left panel, control: *n* = 7; PPDA low dose: *n* = 5; PPDA high dose: *n* = 6). PPDA fully blocks LTD and at cortical pathway at both doses (right panel, control: *n* = 6; PPDA low dose: *n* = 6; PPDA high dose: *n* = 6). **(B)** Summary of the average fEPSP slope over the last 20 min of recording after ppLFS. Insets show I/O curves on top and below representative traces of extracellular field potentials averaged across 10 min before ppLFS (black line) and the last 10 min of recording after ppLFS (gray line). Data represent the mean ± SEM. **p < 0.01, *p < 0.05, ns = non significant.

To determine which other NR2 subunits may be implicated in LTD at the cortical pathway, we next tested the contribution of NR2C/D subunits [NR2A was previously reported not to be involved in LA–LTD (Dalton et al., 2012)]. We used PPDA, a potent and dose-dependent selective NR2C/D antagonist (Hrabetova et al., 2000; Feng et al., 2004). We used PPDA at low concentration (0.25 μM) to preferentially antagonize NR2C/D subunits, and at high concentration (1 μM) to antagonize all NR2 subunits (Feng et al., 2004). At 0.25 μM, PPDA fully blocked LA–LTD specifically at the cortical input, but had no effect at the thalamic pathway (**Figure 4**). In contrast, 1 μM of PPDA abolished LA–LTD at both pathways (thalamic, control: 45.2 ± 8.5%, *n* = 7; PPDA 0.25 μM: 44.3 ± 9.1%, *n* = 5; PPDA 1 μM: 113.6 ± 20.6%, *n* = 6, *p* < 0.01. Cortical, control: 41.6 ± 9.8%, *n* = 6; PPDA 0.25 μM: 113.3 ± 14.1%, *n* = 6, PPDA 1 μM: 107.1 ± 22.2%, *n* = 6, *p* < 0.05, **Figure 4**). I/O curves were not affected by PPDA at either concentration (**Figure 4A**, insets). Overall, these results indicate that LTD at the thalamic pathway depends on NR2B-containing NMDARs, whereas LTD at the cortical pathway depends on NR2C/D-containing NMDARs.

**FIGURE 5:**
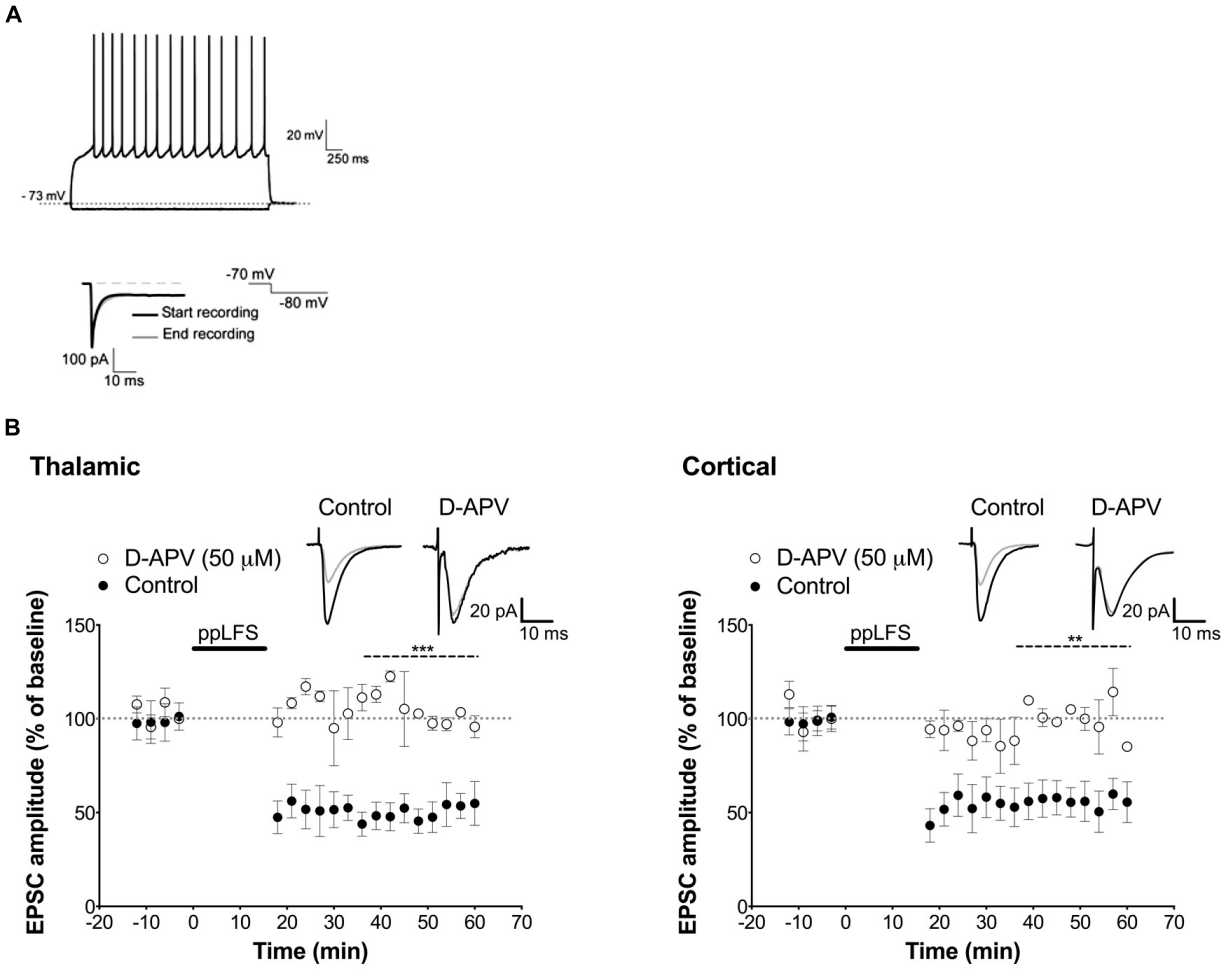
**NMDAR-dependent LTD in single pyramidal cells. (A)** On top, response of a LA cell to current injection of -0.10 and +0.15 nA and below, hyperpolarizing voltage steps of 10 mV from a holding potential of -60 mV were used to measure series resistance. **(B)** D-APV (50 μM) blocks LA–LTD induced at the thalamic pathway (left panel, control: *n* = 4; D-APV: *n* = 3), and at the cortical pathway (right panel, control: *n* = 5; D-APV: *n* = 3). Insets show the average of 10 sweeps of a single cell recorded 10 min before (black) and 30 min after (gray) ppLFS. Data represent mean ± SEM. ***p < 0.001, **p < 0.01.

### DISTINCT LOCI OF LTD INDUCTION AT THALAMIC AND CORTICAL PATHWAYS

Although LTD is generally thought to be induced post-synaptically, it is known that NMDAR-dependent LTD can also occur presynaptically in several brain regions (Casado et al., 2002; Sjostrom et al., 2003; Rodriguez-Moreno and Paulsen, 2008; Rodriguez-Moreno et al., 2010). NR2B-containing NMDARs are mostly localized post-synaptically (Loftis and Janowsky, 2003; Mameli et al., 2005; Miwa et al., 2008; Yu et al., 2010) and NR2C/D-containing NMDARs are mostly presynaptic (Thompson et al., 2002; Mameli et al., 2005; Grilli et al., 2009) and have been implicated in presynaptic LTD in the somatosensory cortex (Banerjee et al., 2009). Because NR2B and NR2C/D subunits are differentially involved in LTD at thalamic and cortical pathways, we hypothesized that LTD may have different loci of induction at thalamic and cortical pathways. We tested this hypothesis using whole-cell patch clamp recording in LA pyramidal neurons. The recorded cells (*n* = 32) showed a firing pattern and spike frequency adaptation characteristic of LA pyramidal neurons (**Figure 5A**; Weisskopf and LeDoux, 1999; Faber et al., 2001). The average resting potential of these neurons was -67.6 ± 4.3 mV. We observed a mono-exponential relationship between current transients and voltage steps, indicating that excitatory cells in LA behave as single electrical compartments (*t*_1_ = 40.65 ± 0.1 ms). Transients were also used to estimate series resistance (15.3 ± 4.23 MΩ), input resistance (235 ± 42.47 MΩ) and membrane capacitance (67.7 ± 16.8 pF), all typical values for LA excitatory cells (Weisskopf and LeDoux, 1999; Faber et al., 2001).

**FIGURE 6:**
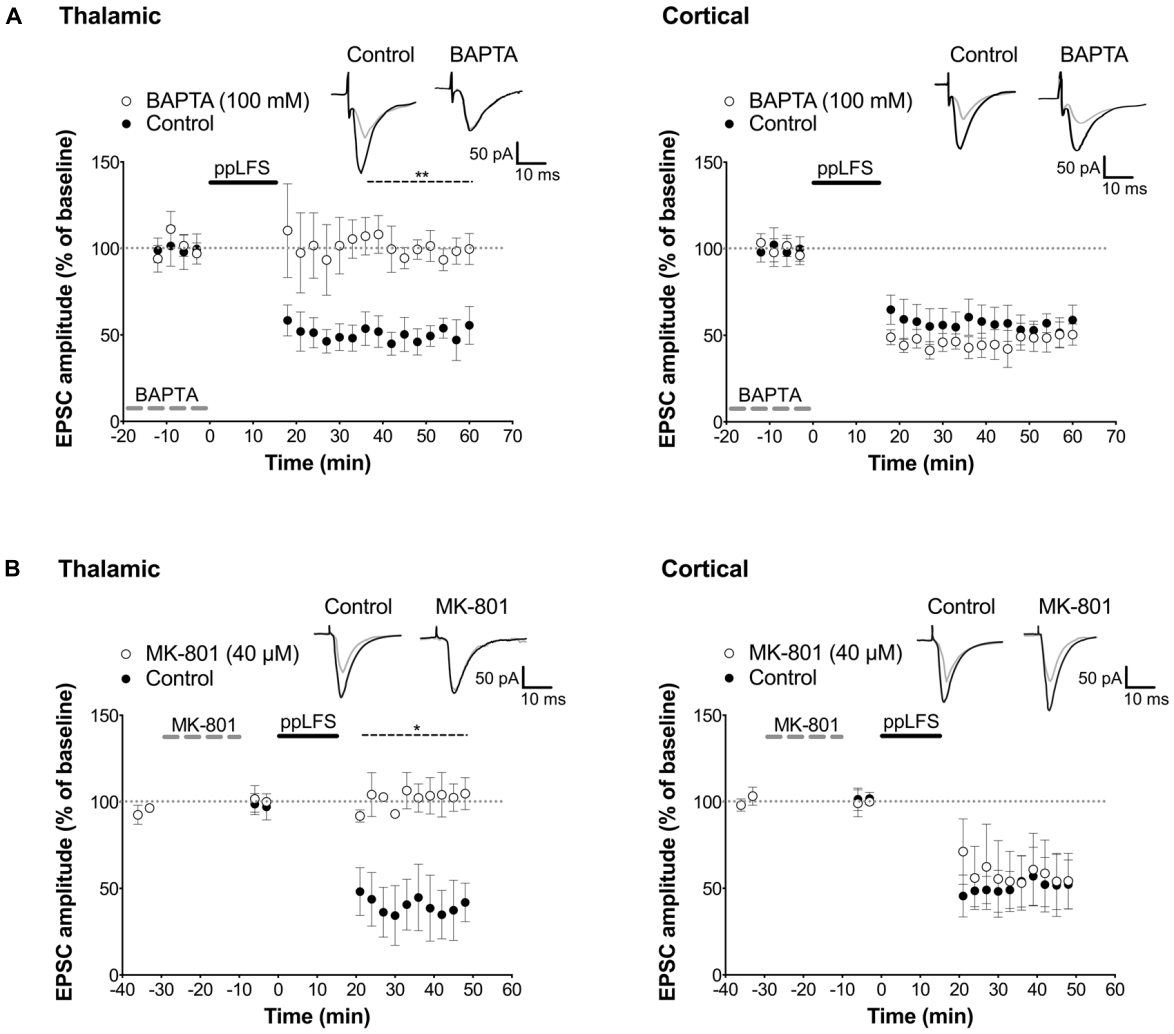
**Distinct locus of LTD induction at thalamic and cortical pathways. (A)** Dialysis of BAPTA blocks LA–LTD induced at the thalamic pathway (left panel, control: *n* = 4; BAPTA: *n* = 4), but not at the cortical pathway (right panel, control: *n* = 5; BAPTA: *n* = 5). Insets show averaged traces of 10 sweeps taken 10 min before (black) and 30 min after (gray) ppLFS. **(B)** Dialysis of MK-801 (40 μM) blocks LA–LTD induced at the thalamic pathway (left panel, control: *n* = 3; MK-801: *n* = 3), but not at the cortical pathway (right panel, control: *n* = 3; MK-801: *n* = 3). Insets show averaged traces of 10 sweeps taken 10 min before (black) and 30 min after (gray) ppLFS. Data represent mean ± SEM. **p < 0.01, *p < 0.05.

Before assessing the locus of LTD induction, we examined whether LTD can be induced in individual excitatory LA neurons with the ppLFS protocol in current clamp configuration, and whether it depends on NMDARs. ppLFS induced a robust and persistent LTD in LA neurons, which was similar at thalamic and cortical inputs (thalamic: 47.38 ± 9.74%, *n* = 4; cortical: 56.2 ± 4.6% *n* = 5, *p* > 0.3, **Figure 5B**). LTD was blocked by D-APV, confirming that it is NMDAR-dependent (thalamic: D-APV: 112.5 ± 3.0%, *n* = 3, *p* < 0.001; cortical: D-APV: 108.1 ± 6.2%, *n* = 3, *p* < 0.01, **Figure 5B**). Because post-synaptic plasticity depends on changes in post-synaptic intracellular calcium concentration, we examined whether LTD is post-synaptic by preventing calcium increase at the post-synaptic site using the membrane impermeable calcium chelator BAPTA (100 mM, dialyzed for 20 min before ppLFS). LTD at the thalamic pathway was fully blocked by BAPTA (control: 47.6 ± 6.7%, *n* = 4; BAPTA: 98.9 ± 5.7%, *n* = 4, *p* < 0.01) but it was not affected at the cortical pathway (control: 52.0 ± 6.6%, *n* = 5, BAPTA: 46.9 ± 6.1%, *n* = 5, *p* > 0.5, **Figure 6A**). These results suggest that the induction of LTD requires a post-synaptic rise in calcium at thalamo-LA synapses but not at cortico-LA synapses. To further assess the synaptic locus of LTD at thalamic and cortical synapses, we selectively blocked post-synaptic NMDARs before LTD induction by intracellular dialysis of the activity-dependent NMDAR antagonist MK-801 (40 μM) into the pyramidal-like LA neuron. In the presence of MK-801, LTD was fully blocked at thalamo-LA synapses (control: 37.9 ± 13.5%, *n* = 3, MK-801: 104.3 ± 5.4%, *n* = 3, *p* < 0.05) but was not affected at cortico-LA synapses (control: 52.5 ± 14.2%, *n* = 3, MK-801: 54.8 ± 11.6%, *n* = 3, *p* > 0.9, **Figure 6B**), suggesting that LTD requires the activation of post-synaptic NMDARs at thalamic but not cortical synapses. Together, these results support a post-synaptic locus of LA–LTD at the thalamic pathway that likely depends on post-synaptic NMDARs, but a mechanism independent of post-synaptic NMDARs and independent of changes in post-synaptic calcium at the cortical pathway.

## DISCUSSION

The protein phosphatases PP2B and PP1 are key players in the regulation of synaptic strength, and in the formation and the maintenance of memory traces (Lisman and Zhabotinsky, 2001; Mansuy and Shenolikar, 2006; Baumgärtel and Mansuy, 2012). Activation of PP2B/PP1 signaling is known to be necessary for LTD in different brain regions (Kirkwood et al., 1993; Mulkey et al., 1994; Morishita et al., 2001; Lin et al., 2003b; Yasuda et al., 2003). This study provides novel evidence that these phosphatases are also involved in the induction of LTD in LA at both thalamic and cortical pathways. This finding is in line with previous results showing that depotentiation at the cortical pathway in LA requires PP2B (Lin et al., 2003a), and that PP2B and PP1 play an important role in memory tasks that depend on the amygdala including conditioned taste aversion and extinction of fear memory (Lin et al., 2003b; Baumgärtel et al., 2008; Oberbeck et al., 2010; Koshibu et al., 2011). They also complement findings in the hippocampus that PP2B or PP1 inhibition enhances hippocampal LTP and memory performance in hippocampus-dependent tasks (Malleret et al., 2001; Genoux et al., 2002) but impairs LTD (Jouvenceau et al., 2006). Taken together, these findings support the concept that PP2B/PP1 are key regulators of synaptic plasticity, and that their inhibition favors LTP and memory acquisition, but impairs LTD and memory extinction in both hippocampus and amygdala.

Our finding that LTD at the thalamic LA pathway is NR2B-dependent is consistent with previous studies (Müller et al., 2009; Dalton et al., 2012; Park et al., 2012). NR2B is present in post-synaptic densities (PSD) in LA (Miwa et al., 2008), and LTD at the thalamic pathway depends on NR2B-dependent post-synaptic AMPAR endocytosis (Yu et al., 2010). Surprisingly, we observed that LTD induced at the cortical pathway is independent of NR2B signaling, since NR2B antagonists do not block LTD induction. Instead, we observed that blocking NR2C/D subunits fully prevents LTD at the cortical pathway, but does not affect LTD at the thalamic pathway. The observation that LTD at the cortical pathway is NR2B-independent contrasts with a previous report showing that antagonizing NR2B blocks LTD at both pathways in horizontal slices from adult mice (Müller et al., 2009). This apparent discrepancy likely results from a different orientation of the slices leading to different sites of stimulation and recording. Specifically, placing the stimulating electrode laterally to the internal capsule in coronal slices primarily activates cortical afferents to LA, but in horizontal slices, it also activates afferents from the entorhinal and perirhinal cortex (von Bohlen und Halbach and Albrecht, 2002; Müller et al., 2009). The spatial organization of excitatory and inhibitory connections within the LA depends as well on slice orientation (Samson et al., 2003; Samson and Pare, 2006). It thus needs to be determined whether LTD differentially relies on NR2B or NR2C/D-containing receptors in the cortical pathway depending on the slice orientation. Given our clear finding that thalamic and cortical input to LA rely on different molecular and post-synaptic mechanisms, we postulate that projections to LA from the perirhinal and entorhinal cortex likely engage different mechanisms as well. Notably, most electrophysiological studies in the amygdala are conducted in coronal sections rather than horizontal sections (Huang and Kandel, 1998; Heinbockel and Pape, 2000; Tsvetkov et al., 2002; Humeau et al., 2003; Hong et al., 2009; Dalton et al., 2012). To our knowledge, this is the first report showing in coronal slices, a strong and reproducible induction of LTD at cortical afferents to LA by low-frequency stimulation, without the need of prior potentiation (Hong et al., 2009). As highlighted by Müller et al. (2009) this demonstrates that previous lack of LTD at cortical afferents (Tchekalarova and Albrecht, 2007; Pape and Pare, 2010) may be due to inadequate protocols for that specific pathway rather than an intrinsic failure to decrease synaptic transmission at cortical inputs to the LA. The availability of a robust LTD induction protocol at both input pathways to the amygdala in coronal slices shall allow further analyses of the mechanisms of LTD regulation in the amygdala.

Our observation that different NR2 subunits mediate the effects of ppLFS-induced LTD at both input pathways to the LA are in agreement with previous studies reporting differences in the molecular cascades at these pathways in LTP and depotentiation (Humeau et al., 2003, 2005; Hong et al., 2009; Jung et al., 2010; Meis et al., 2012). Although the distribution of NMDAR subunits in the amygdala remains largely unknown, the receptor kinetics at resting membrane potential is known to be different at cortical and thalamic pathways (Weisskopf and LeDoux, 1999). NMDARs at cortical inputs are less sensitive to magnesium blockade than at thalamic inputs, and the kinetic properties are akin to NR2C/D-containing NMDARs at the cortical pathway, but resemble NR2A/B-containing NMDARs at the thalamic pathway (Monyer et al., 1994; Weisskopf and LeDoux, 1999; Cull-Candy et al., 2001). This is in agreement with our observation that NR2C/D-containing receptors seem to mediate LTD at the cortical pathway, but not at the thalamic pathway.

Presynaptic NR2C/D-containing NMDARs are believed to be involved in spike-timing dependent LTD in the cortex (Banerjee et al., 2009). This prompted us to investigate the site of LTD induction at both LA pathways by whole cell patch-clamp recording. We observed that LTD induction occurs post-synaptically at the thalamic pathway, but is independent of post-synaptic calcium influx or post-synaptic NMDARs at the cortical pathway. These findings for LTD complement previous reports for LTP in the amygdala showing that LTP engages different pre- and post-synaptic mechanisms at thalamic and cortical pathways (Huang and Kandel, 1998; Humeau et al., 2003, 2005, 2007; Tsvetkov et al., 2004; Rumpel et al., 2005; Shaban et al., 2006).

Although distinct NMDAR subunits and post-synaptic mechanisms are involved at thalamic and cortical afferents to the LA, both pathways converge onto a PP2B/PP1 signaling cascade. In the hippocampus, calcium influx through NMDARs, rather than other calcium channels, is specifically required for PP2B and PP1 activation (Unoki et al., 2012). It is possible that presynaptic calcium influx through NR2C/D-containing NMDARs, and post-synaptic calcium influx through NR2B-containing NMDARs, lead to the activation of PP2B/PP1 at the cortical and thalamic pathway, respectively, a possibility that will need to be tested in future experiments. Whether NR2C or NR2D subunits are localized presynaptically at cortical but not at thalamic afferents to LA will also need to be determined, as well as the molecular mechanisms downstream of PP2B/PP1 activation at both pathways. In the hippocampus, PP2B and PP1 have presynaptic and post-synaptic targets (Strack et al., 1999; Nakano-Kobayashi et al., 2007; Baumgärtel and Mansuy, 2012). In hippocampal and cortical neurons, PP1 can dephosphorylate NR2B (Farinelli et al., 2012; Prabhu Ramya et al., 2012), resulting in a downregulation of NMDAR activity (Farinelli et al., 2012). Similarly, in cerebellar granule cells, PP2B downregulates NR2C expression (Suzuki et al., 2005), thus it is possible that PP2B/PP1 dephosphorylate NR2B and NR2C subunits differentially in LA in response to LTD induction. Finally, the contribution of other receptors such as metabotropic glutamate receptors (mGluRs) in LA LTD cannot be excluded. Group I mGluRs have previously been shown to contribute to ppLFS-induced depotentiation at the thalamic pathway (Kim et al., 2007), while presynaptic group II mGluRs seem to be involved at cortical afferents (Hong et al., 2009). Although mGluR-dependent LTD appears to involve tyrosine phosphatases rather than serine/threonine phosphatases such as PP2B and PP1 (Lin et al., 2005; Collingridge et al., 2010), they may also contribute to the differential molecular effects of ppLFS-induced LTD at both pathways.

LA–LTD is associated with the extinction of fear memory (Kim et al., 2007; Hong et al., 2009; Park et al., 2012). Since weakening and erasure of traumatic memory traces is critical for the management of anxiety disorders including PTSD (Kindt et al., 2009; Monfils et al., 2009; Quirk et al., 2010; Holzschneider and Mulert, 2011; Pitman et al., 2012), understanding the molecular mechanisms of LTD in the amygdala has important clinical implications. Our findings highlight the potential of therapeutically targeting PP2B/PP1 signaling to facilitate fear extinction learning in anxiety-related disorders (Baumgärtel et al., 2008; Bahi et al., 2009; Koshibu et al., 2011).

## Conflict of Interest Statement

The authors declare that the research was conducted in the absence of any commercial or financial relationships that could be construed as a potential conflict of interest.
